# Editorial: Soilborne pathogenic fungi: systematics, pathogenesis and disease control

**DOI:** 10.3389/fmicb.2024.1525583

**Published:** 2024-12-06

**Authors:** Shitou Xia, W. G. Dilantha Fernando, Junxing Lu

**Affiliations:** ^1^Hunan Provincial Key Laboratory of Phytohormones and Growth Development, College of Bioscience and Biotechnology, Hunan Agricultural University, Changsha, China; ^2^Department of Plant Science, University of Manitoba, Winnipeg, MB, Canada; ^3^Chongqing Key Laboratory of Plant Environmental Adaptations, College of Life Science, Chongqing Normal University, Chongqing, China

**Keywords:** soilborne diseases, fungal pathogenesis, phylogenomics, plant-fungal interactions, biocontrol agents

Soilborne diseases, frequently triggered by a spectrum of pathogens such as *Sclerotium rolfsii, Rhizoctonia solani*, and *Fusarium oxysporum* etc., inflict considerable damage on a variety of crops. The damage manifests as wilting, stunting, chlorosis, and, in severe cases, plant death. These diseases pose a formidable management challenge due to the pathogens' resilience in the soil in the absence of a host. Gaining insights into their survival mechanisms, dispersal patterns, and interactions with host plants is essential for crafting effective control strategies. Recent strides in molecular biology and genomics have shed light on these interactions, clearing a path for environmentally friendly biocontrol methods that safeguard crops and minimize yield loss. The selected articles published in this Research Topic include four research articles and three reviews.

Umar, Elshikh et al. and Umar, Yuan et al. present two groundbreaking studies that offer profound insights into the fungal pathogens within the *Ganoderma* genus. The first study explores the competitive antagonism between *Trichoderma* species and the newly identified wood pathogen *Ganoderma camelum*, underscoring the role of laccase in their interaction (Umar, Elshikh et al.). The second one reveals a novel species, *Ganoderma segmentatum*, and its pathogenic relationship with *Vachellia nilotica* (Umar, Yuan et al.). These papers highlight the critical importance of understanding fungal ecology and pathogenicity, which is vital for devising strategies to alleviate the impact of these fungi on agriculture and forestry. Further investigation into the utilization and interaction of these species is necessary for devising more effective control measures. Collectively, these findings highlight the pivotal role of genomics in advancing research on fungal diseases and bolstering sustainable agricultural practices.

Sayari et al. provide a deep dive into the genomics era's impact on our understanding of *Verticillium* species, a group of fungi that pose significant agricultural challenges. The authors skillfully illuminate how genomic approaches have unraveled the complexity of these pathogens, offering new understanding of their pathogenicity, virulence, and the mechanisms underlying host resistance. By meticulously summarizing recent advancements, the review emphasizes the crucial role of genomics in nurturing sustainable agricultural solutions through disease control strategies and developing resistant crop varieties. The integration of comparative genomics, population genomics, and functional genomics techniques positions this review as an invaluable resource for researchers. It underscores the continuous evolution of disease management in the genomics era.

Ma et al. contribute significantly to plant pathology with their comprehensive analysis of *Fusarium* species causing crown rot in wheat in Shandong province, with a particular focus on the dominance of *F. pseudograminearum*. Identifying novel pathogens, such as *F. incarnatum* and *F. ipomoeae*, expands our current understanding of *Fusarium*'s impact on wheat production. This research also lays the foundation for developing targeted strategies to counter the threat of *Fusarium* crown rot to wheat cultivation. In addition, the study could benefit from a broader sampling range, longer-term data collection, and an investigation into the effectiveness of various fungicides against identified *Fusarium* species.

Zhu et al. deliver a timely and comprehensive analysis of the virulence factors of *Sclerotinia sclerotiorum*, a devastating broad host range plant pathogen. By elucidating the role of oxalic acid, cell wall degrading enzymes, and effector proteins in pathogenicity, this paper enhances our understanding of *S. sclerotiorum*'s infection mechanisms. The exploration of host-induced gene silencing (HIGS) as a potential control strategy found that this mechanism can be effective, offering an avenue for managing this challenging pathogen and enhancing crop resistance. This work will provide essential reference for researchers and agronomists seeking sustainable solutions to *Sclerotinia*-related crop losses.

Bandara and Kang meticulously investigate the impact of *Trichoderma virens* application methods on tomato growth and *rhizomicrobiome*, offering critical insights for biocontrol strategies. By elucidating how different application timings of *T. virens* influence plant health and soil suppressiveness against *Fusarium oxysporum*, this research advances our understanding of microbial-induced disease resistance. It is suggested that the at-transplant application promotes tomato growth and soil pathogen suppression, highlighting a promising approach for sustainable agriculture. This study paves the way for further investigations into optimizing biocontrol agent applications in various production systems.

Trenk et al. offer a timely and comprehensive analysis of pea root rot disease, a pervasive and devastating problem in sustainable pea cultivation. By highlighting the multifaceted nature of the disease and underscoring the significance of recent diagnostic advancements, the authors set the stage for a new era in disease management. The focus on an integrative approach that encompasses genetic resistance, soil microbiome modulation, and advanced diagnostics not only underscores the complexity of the challenge but also opens avenues for developing resilient pea varieties.

These articles presented in this Research Topic offer a comprehensive exploration of soilborne diseases. The studied cases underscore the intricacy of soilborne pathogens and provide practical insights for the development of sustainable agricultural practices that can counteract these pervasive diseases. Therefore, the topic collectively underlines the importance of adopting an integrated approach to address these challenges and paves the way for future advancements in plant pathology and biocontrol strategies.

